# Seizures, Epilepsy, and NORSE Secondary to Autoimmune Encephalitis: A Practical Guide for Clinicians

**DOI:** 10.3390/biomedicines11010044

**Published:** 2022-12-25

**Authors:** Alberto Vogrig, Gian Luigi Gigli, Annacarmen Nilo, Giada Pauletto, Mariarosaria Valente

**Affiliations:** 1 Clinical Neurology, Santa Maria della Misericordia University Hospital, Azienda Sanitaria Universitaria Friuli Centrale (ASU FC), 33100 Udine, Italy; 2 Department of Medicine (DAME), University of Udine Medical School, 33100 Udine, Italy; 3 Neurology Unit, Santa Maria della Misericordia University Hospital, Azienda Sanitaria Universitaria Friuli Centrale (ASU FC), 33100 Udine, Italy

**Keywords:** limbic encephalitis, paraneoplastic neurological syndromes, FIRES, EEG, ketogenic diet

## Abstract

The most recent International League Against Epilepsy (ILAE) classification has included “immune etiology” along with other well-known causes of epilepsy. This was possible thanks to the progress in detection of pathogenic neural antibodies (Abs) in a subset of patients, and resulted in an increased interest in identifying potentially treatable causes of otherwise refractory seizures. Most autoimmune encephalitides (AE) present with seizures, but only a minority of cases evolve to long-term epilepsy. The risk of epilepsy is higher for patients harboring Abs targeting intracellular antigens (T cell-mediated and mostly paraneoplastic, such as Hu, CV2/CRMP5, Ma2, GAD65 Abs), compared with patients with neuronal surface Abs (antibody-mediated and less frequently paraneoplastic, such as NMDAR, GABAbR, LGI1, CASPR2 Abs). To consider these aspects, conceptual definitions for two entities were provided: acute symptomatic seizures secondary to AE, and autoimmune-associated epilepsy, which reflect the different pathophysiology and prognoses. Through this manuscript, we provide an up-to-date review on the current state of knowledge concerning diagnosis and management of patients with Ab-mediated encephalitis and associated epilepsy. Special emphasis is placed on clinical aspects, such as brain magnetic resonance imaging (MRI) and cerebrospinal fluid (CSF) specificities, electroencephalographic (EEG) findings, cancer screening and suggestions for a rational therapeutic approach.

## 1. Introduction

Although epilepsy is one of the more frequent neurologic disorders worldwide, affecting approximately 1% of the world population, in 1/3 of the patients the etiology of the seizure disorder remains unknown [1,2]. Recently, there has been accumulating evidence to support the key role of neuroinflammation in triggering or sustaining epileptic activity, especially in patients who present with seizures refractory to antiseizure medications (ASMs) in the context of an antibody (Ab)-mediated encephalopathy [3,4]. As a result, the most recent International League Against Epilepsy (ILAE) classification has included “immune etiology” along with the other well-known causes of epilepsy (structural, genetic, infectious, and metabolic) [5], paving the way for new therapeutic options in this difficult-to-treat group of patients, considering that seizures in this setting often respond dramatically to immunotherapy [3,4]. 

Despite the high frequency of seizures and propensity to develop status epilepticus (SE) in the acute stage of autoimmune encephalitis (AE), most patients with AE do not develop an enduring predisposition to seizures over the long-term [3,6]. This important concept was highlighted by the ILAE in 2020, when the Autoimmunity and Inflammation Taskforce proposed two main diagnostic entities: “acute symptomatic seizures secondary to AE” and “autoimmune-associated epilepsy”, the latter of which applied to a minority of cases, often due to the development of structural abnormalities after inflammation vanishes (e.g., mesial temporal sclerosis) or an enduring antigenic trigger (e.g., cancer in paraneoplastic cases) [7]. The amount of new information in this field in the last decade in terms of clinical specificities, laboratory diagnostics and treatment options has made it difficult for neurologists to approach patients with AE and seizures. 

The aim of this review is to provide a comprehensive and practical overview on diagnosis and management of immune-mediated seizures and epilepsy.

## 2. Search Strategy and Selection Criteria

References for this narrative review were identified by searches of PubMed/MEDLINE between January 2000 and August 2022, focusing mostly on recent (last 5 years) publications concerning the diagnosis and treatment of seizures in the context of AE, and references from relevant articles. The search terms “autoimmune encephalitis” and “epilepsy”, “autoimmune encephalitis” and “seizures”, “autoimmune epilepsy”, “immune-mediated epilepsy”, “autoimmune encephalitis” and “EEG” (electroencephalogram) were used. The literature search was limited to the English language, and the final reference list was obtained on the basis of relevance to the topics covered in this review.

## 3. Autoimmune and Paraneoplastic Encephalitis: General Concepts

Any inflammatory disorder primarily affecting the brain (e.g., acute disseminated encephalomyelitis (ADEM), Rasmussen encephalitis, etc.) or systemic inflammatory disorder that also targets the central nervous system (e.g., sarcoidosis or systemic lupus erythematosus) can potentially trigger epileptic seizures, but in this review we will focus on a highly paradigmatic group of brain diseases known as autoimmune encephalitides. 

The initial description of these disorders stems from the clinicopathologic study, performed by John Arthur Nicholas Corsellis in the 1960s, of an “unidentified cerebral illness” predominantly involving the limbic system in patients with an underlying systemic carcinoma. He defined this condition as “limbic encephalitis” (LE) and specified that the clinical presentation included weight loss, seizures, confusion and complete amnesia [8]. The link between the two apparently unrelated conditions (limbic encephalitis and cancer) was subsequently demonstrated by the presence of auto-Abs targeting proteins expressed by both the nervous system and the neoplastic cells, therefore named “onconeural” [9]. The onconeural Ab spectrum has steadily grown since the description of Tr antibodies in the 1970s [10] (usually not associated with seizures but most commonly with a cerebellar disorder [11]), Hu Abs in the 1980s [12], CV2 Abs in the 1990s [13] (both strongly associated with LE and other multifocal neurological syndromes with an increased prevalence of seizures) and many others later on, including Abs identified nowadays (e.g., KLHL11 Abs in 2019 [14,15]). The intracellular location of these antigens implies that onconeural Abs are not directly pathogenic (since they cannot reach their target) but can serve as reliable biomarkers of the immune-mediated disorder [9]. Conversely, a T-cell mediated pathogenesis is advocated as the cause of an irreversible neuronal injury that explains the poor response to immunotherapy observed in most patients with paraneoplastic LE (with rare exceptions, such as anti-Ma2 encephalitis, which can be treatment responsive in a non-negligible number of cases) [9,16]. 

**Figure 1:**
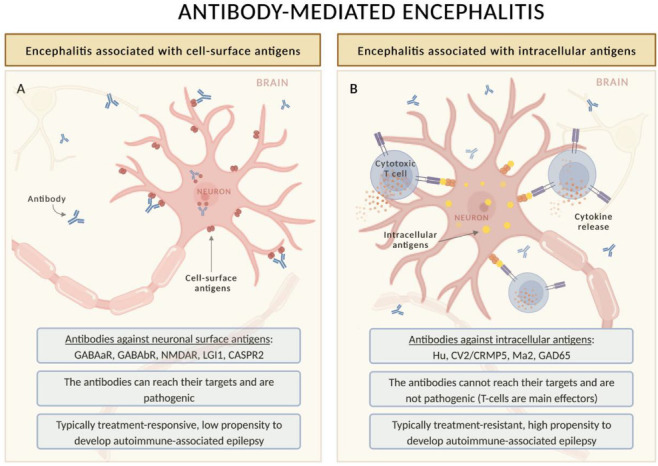
Pathophysiology of autoimmune encephalitis and its relationship with the risk of autoimmune-associated epilepsy. The diagram focuses on forms of autoimmune encephalitis commonly presenting with acute symptomatic seizures. In encephalitis associated with antibodies targeting cell surface antigens, the antibodies have access to their targets and can alter the structure and function of the antigen (**A**). This alteration is usually treatment responsive, especially at early stages. Therefore, few patients develop autoimmune-associated epilepsy. Conversely, in encephalitis associated with antibodies against intracellular epitopes, the antibodies cannot reach the intracellular antigens, and cytotoxic T-cell mechanisms are mainly involved (**B**). This process can lead to neuronal loss, and it is therefore less treatment responsive: many patients develop autoimmune-associated epilepsy.

Still, today, as in the Corsellis’ description, the diagnostic criteria of LE include short-term memory loss, seizures, and psychiatric manifestations rapidly progressing in less than 3 months [17]; however, the historical concept of LE as a phenotype typically associated with cancer has changed dramatically in the last decade [18]. This paradigm shift followed the description of LGI1-Ab encephalitis [19], now considered to be the most frequent form of LE [20], which is typically non-paraneoplastic and almost always associated with seizures [3,21]. This Ab belongs to a family that targets cell-surface or synaptic proteins, which includes NMDAR (the first of the group to be discovered by Dalmau and collaborators in 2007 [22]), but also AMPAR, GABAaR and GABAbR, among others [9]. Some of these Abs are associated with isolated or predominant limbic involvement, while others (e.g., NMDAR) are associated with a more widespread (limbic and extra-limbic) form of autoimmune encephalitis [23]. Neuronal surface Ab-mediated encephalitides are different in several aspects compared with encephalitides associated with onconeural Abs (Figure 1): (i) the association with cancer is less strong, with the proportion of paraneoplastic cases depending on the type of Ab as well as demographic features of the patients (e.g., age and smoking habit), (ii) neuronal surface Abs are pathogenic, and (iii) the response to treatment is usually more satisfactory [9]. Despite these differences, it is important to note that paraneoplastic and non-paraneoplastic cases may be clinically indistinguishable, justifying a comprehensive tumor search in all patients with AE [18]. 

Autoimmune and paraneoplastic encephalitides are rare disorders, with an incidence rate of antibody-positive AE (3.6 per million person years in Rhône-Ain-Isère region, France [20] vs. 4 per million person years in Olmsted County, USA [24]) and PNS (4.1 per million person years in France [20] vs. 6 per million person years in Olmsted County, USA [25] vs. 8.9 per million person years in Friuli Venezia Giulia, Italy [26]) that is low, but similar to that of infectious encephalitides [24,26]. This means that before making a diagnosis of AE as the cause of seizures and epilepsy, several more common alternative diagnoses need to be ruled out, including structural, genetic, infectious, and metabolic etiologies [9]. This is particularly important to avoid misdiagnoses and the inappropriate initiation of immunotherapies. For instance, in a National Reference Center, approximately 2% of patients referred for suspected AE were finally diagnosed with glioblastoma [27]. Diagnosis of AE (as well as exclusion of AE-mimics) requires paraclinical tests, in particular brain magnetic resonance imaging (MRI) and cerebrospinal fluid (CSF) analysis, which typically show inflammatory alterations (see below), but can sometimes be normal [3] (especially in elderly patients with LGI1, CASPR2 and IgLON5-Ab syndromes [28], the latter of which is atypical because it does not associate with seizures and has a different immunopathogenesis [29]).

AE is defined by clinical criteria which comprise the subacute onset of memory deficits, altered mental status, and psychiatric disorders in association with either new focal findings, seizures, CSF pleocytosis or findings consistent with encephalitis on brain MRI [17]. A distinct set of criteria have been developed in children to account for different clinical presentations, which can be acute or subacute; well-defined syndromes may be lacking and the clinical picture may include focal or diffuse neurological deficits, including more insidious presentations, such as developmental regression [30]. The most frequent neuronal surface Abs associated with seizures and epilepsy are shown in Table 1.

Several advances have also been made in the field of paraneoplastic disorders, where a new set of diagnostic criteria have been recently developed to enhance the clinical care of patients and to increase diagnostic specificity [18]. The consistency between neurological phenotype, antibody, and cancer was highlighted so as to (i) avoid spurious clinical associations and (ii) ensure that the link with cancer is causal and not only chronological [18]. Using a novel tool (PNS-Care score) which comprises three items (clinical phenotype, antibody type, and the presence of cancer that needs to be consistent with the other items), patients can be diagnosed with PNS on three levels of certainty (possible, probable, and definite). Consistency in the oncological association is particularly relevant, as some cancers are highly prevalent in elderly populations (e.g., prostate cancer in elderly men) and their presence does not necessary correlate with a new-onset neurological syndrome [18]. In fact, most PNS develop (and often antedate) an underlying tumor belonging to few categories: lung (more frequently small-cell lung cancer, but also non-small-cell lung cancer), breast, ovarian cancer or lymphoma [9,26,31]; however, they are infrequent in other tumor types (e.g., gastrointestinal, bladder or renal cancers [32]). The most common Abs against intracellular antigens associated with epileptic manifestations are shown in Table 2. 

Recently, it was observed that patients with cancer treated with immunotherapy (i.e., immune checkpoint inhibitors, ICIs) can develop severe neurological complications in approximately 1–3% of cases, most often peripheral nervous system disorders (especially myositis, myasthenic syndromes, and overlap syndromes including myocarditis) [33,34]. A minority of cases (especially those with associated lung cancer and onconeural Abs) may develop central nervous system complications, which can manifest as LE, meningoencephalitis, or cerebellitis [35]. AE triggered by ICIs can manifest with seizures in about 30% of the cases [36], with higher frequencies in cases with limbic involvement (37% [35] and 38% [37] in two different studies). However, this proportion of seizures is lower than that reported in a systematic review focused on AE not related to ICIs, where about 70% of patients had seizures during their illness and 85% had EEG abnormalities [38]. Seizures seem to be more common in younger patients with AE [38]. 

On clinical grounds, few seizure types are pathognomonic of an autoimmune etiology, including faciobrachial dystonic seizures (FBDS) [21] and seizures that arise from perisylvian (insular-opercular) regions [39]. FBDS are very brief (<3 s) tonic muscle contractions of the arm (appearing as dystonic posturing) and face (as a grimace) and less frequently involving the leg (which can lead the patient to fall). They are usually unilateral, but can independently affect both sides asynchronously and can occur up to 100 times a day, including during sleep [3]. FBDS can manifest alone or be accompanied (generally followed) by alteration of awareness or automatisms (including rare forms such as ictal drinking), probably suggesting seizure spreading to the mesial temporal lobe [40]. These seizures are considered pathognomonic of anti-LGI1 encephalitis, and their early identification (and consequent start of immunotherapy, in particular corticosteroids) can avoid the onset of cognitive (especially memory) dysfunction characteristic of the disease [21]. 

Seizures with perisylvian semiology, including autonomic (flushing, palpitations, and piloerection) and somatosensory/viscerosensory (paresthesia restricted to the head, face, or perioral areas, sensation of numbness or constriction in pharyngeal region, auditory phenomena) symptoms, conversely, are not associated with a specific Ab, but are often indicative of an immune-mediated etiology [39]. 

Prompt recognition of an autoimmune origin of seizures has relevant therapeutic implications. Because of this, several criteria and scoring systems for autoimmune seizures and epilepsy have been proposed (e.g., autoantibody prevalence in epilepsy (APE) score [41], and its subsequent revision (APE2) [42]; antibody contributing to focal epilepsy signs and symptoms (ACES) score [43], etc.). All these scores focus on few items: (i) new onset, drug-resistant seizures (that is, seizures that persist after an adequate trial of two ASMs); (ii) associated clinical features (cognitive symptoms, behavioral changes, autonomic symptoms, language disorders, autoimmune diseases); (iii) evidence of brain inflammation on CSF, MRI or brain biopsy. These criteria are useful, especially in full-blown AE; however, in the most challenging clinical settings (patients with isolated seizures disorder lacking paraclinical signs of inflammation) they do not help in making a correct diagnosis, which still relies on clinical acumen. 

## 4. MRI

Brain MRI is an invaluable paraclinical test which aids in the differential diagnosis and often shows inflammatory alterations in patients with AE. These typically include hyperintensities highly restricted to limbic regions on T2-weighted imaging (as in LE) [9]. Contrast-enhancing lesions are rare but possible (e.g., in 30–40% of patients with anti-Ma2 encephalitis) [16,44]. 

Several neuroimaging specificities are observed in the different Ab-mediated syndromes. For example, brain MRI shows unilateral or bilateral mesial temporal lobe hyperintensities in most cases of anti-LGI1 encephalitis; however, it can be normal in up to 25% of patients [45,46]. In addition, some patients (especially those experiencing FBDS) may show increased T1 or T2 signal abnormalities in the basal ganglia (often contralateral to the tonic-dystonic movements) [47]. Approximately half of patients with anti-GABAbR encephalitis manifest increased fluid-attenuated inversion recovery (FLAIR) signals confined to one or both mesial temporal lobes, while in the other half of the cases brain MRI is normal [48,49,50]. Neuroimaging changes in anti-GABAaR encephalitis are distinctive and similar to those observed in ADEM, characterized by multiple, cortical-subcortical, FLAIR MRI changes and predominantly involving the temporal and frontal lobes [51]. Neuroimaging of anti-NMDAR encephalitis is different from other types of AE—in which brain MRI is often informative—as it is frequently normal, or only non-specific changes are observed (half of the cases), including FLAIR changes in the temporal lobe, cerebral cortex (where contrast enhancement is also possible in early stages), brainstem, basal ganglia and cerebellum (where irreversible atrophy can develop in later stages) [3,23]. Demyelinating lesions are sometimes observed due to overlapping anti-MOG autoimmunity [52]. 

Regarding paraneoplastic cases, patients with anti-Hu autoimmunity often present with clinical signs of dysfunction at multiples sites of the nervous system, including peripheral involvement at the level of dorsal root ganglia, peripheral nerve or nerve roots, as in the so-called paraneoplastic encephalomyelitis [18]. Approximately 10–20% of patients show clinical and MRI involvement of the limbic regions [12,53], and these cases tend to develop both acute symptomatic seizures and autoimmune-associated epilepsy [7] (the destructive T-cell reaction often causes the development of atrophy and enduring predisposition to seizures) [9]. Patients with anti-Ma2 encephalitis may show limbic involvement on brain MRI which is generally accompanied by changes involving the hypothalamic and brainstem regions, which explains their propensity to develop additional symptoms such as narcolepsy, hypersomnia, weight gain, diplopia and supranuclear gaze palsies [16,54]. Interestingly, it was demonstrated that both anti-Hu and anti-Ma2 syndromes can also be triggered by ICIs [55,56].

## 5. CSF Analysis and Antibody Testing

As for brain MRI, CSF examination is important for both confirming the diagnosis and excluding mimics, especially infectious causes of encephalitis; it is also important for ruling out carcinomatous meningitis in patients with an underlying cancer (in both scenarios, a reduction of CSF glucose content should alert the clinicians to the possibility of these alternative etiologies) [9,18]. CSF-inflammatory alterations are not specific for AE, but are often detected. These include pleocytosis and increased protein concentration as well as the presence of CSF-restricted oligoclonal bands (OCBs) [9]. It is important to consider that AE with Abs against NMDAR, GABAbR and AMPAR show frequent inflammatory CSF changes, which can be absent in CASPR2, LGI1 and GABAaR encephalitis [57], accounting for the difficulties in reaching a correct diagnosis. 

CSF collection is also highly important in searching for neuronal Abs, especially for antibodies against surface antigens [3,18]. To improve antibody detection, CSF and serum should be tested at the same time (the sensitivity of one or the other samples depends on the type of Ab, being greater in the CSF for NMDAR, AMPAR, and GABAbR, while greater in serum for onconeural and LGI1 Abs) [3,58]. Positive results obtained by commercial line blots or CBAs need to be confirmed by brain immunohistochemistry (with very few exceptions, such as anti-glycine Abs, in which immunohistochemistry is not useful) [3,18]. 

As always, clinical judgement is of paramount importance, as indiscriminate testing increases the chances of both false-positive and false-negative results [18]. This means that physicians should always critically evaluate positive Ab results if incongruent with the neurologic phenotype and/or oncological associations [18]. In this regard, obtaining an expert opinion of reference centers can be extremely valuable in complex cases [3,18]. Despite appropriate and complete Ab testing, about 7% of LE cases remain seronegative [59].

## 6. EEG Findings

EEG plays a relevant role in the management of AE. In particular, it is widely accepted that prolonged EEG recordings have a crucial role, not only in detecting and managing clinical and subclinical seizures/SE, but also in evaluating the seizure burden in the acute stage (which are sometimes difficult to distinguish from atypical movement disorders as in anti-NMDAR encephalitis), and the long-term risk of epilepsy [60]. Investigations about the diagnostic value of EEG have been mainly focused in anti-NMDAR and anti-LGI1 encephalitis [3]; limited evidence is currently available on other rare forms as well as seronegative cases [3,60]. A small retrospective study published in 2016 found no electrographic differences on routine EEG between patients with Ab-positive and those with seronegative encephalitis [61]. More than 75% of patients with AE present some EEG abnormalities, but normal EEGs are possible [3,60]. A sustained slowing of posterior dominant rhythm on routine EEG has been associated with AE [62], although it can be observed only in one third of AE during the acute phase [60]. In their study of 64 patients, Moise and colleagues found a specific EEG pattern only in cases of anti-NMDAR encephalitis; meanwhile, in other AE subtypes, no specific pattern was identified [63]. Lateralized rhythmic delta activity (16%), lateralized (22%) and/or generalized (16%) periodic discharges, and bilateral independent periodic discharges (3%) were reported without specific Ab association [63].

In anti-NMDAR encephalitis, EEGs have high sensitivity (96%) [64] and should be performed in all suspected cases. Importantly, it can be helpful in a differential diagnosis with schizophrenia [65] (which usually lacks marked EEG changes) in those rare cases with isolated psychiatric manifestations [64]. Three typical EEG patterns have been described with a distinctive chronological order over the course of the disease in anti-NMDAR encephalitis: (1) excessive beta activity at 14–20 Hz, predominant on frontal regions; (2) extreme delta brush, characterized by a continuous delta activity superimposed with fast activity in the beta range, with a synchronous and symmetrical frontal distribution; (3) generalized delta activity, characterized by a diffuse, synchronous and rhythmic delta activity, presented in the advanced stage of disease and often accompanied by abnormal movements [3,66]. 

**Figure 2:**
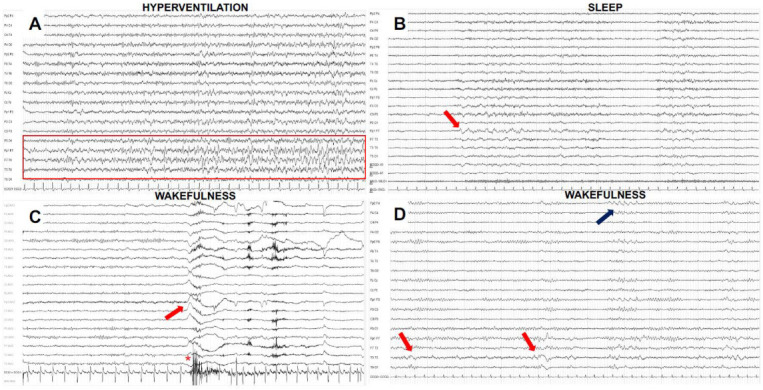
Electroencephalographic findings in patients with antibody-positive autoimmune encephalitides. (**A**) Electrical seizure triggered by hyperventilation in a male patient with anti-LGI1 encephalitis. Its onset is best appreciated with left temporal theta–delta sharp abnormalities that evolve in terms of frequency and location, showing contralateral diffusion. (**B**) The same patient of (**A**): during sleep (N2-NREM phase) shows the presence of lateralized fronto-temporal delta slowing (red arrow). (**C**) Female patient with anti-LGI1 encephalitis: EEG demonstrates a focal slow wave on frontal electrodes (red arrow) preceding faciobrachial dystonic seizure (see muscle artifacts on electrocardiogram channel *). (**D**) Awake EEG in a male patient with anti-GABAbR encephalitis shows intermittent lateralized left temporal delta slowing (red arrows) with anterior and contralateral diffusion (blue arrow). *Parameters of EEG recordings: figures A, B, D bipolar 10/20 standard international montage; high frequency filter: 1.0 Hz; low frequency filter: 70 Hz; notch filter ON; sensitivity 100 microV/mm; 15 mm/s; figure C average reference montage; high frequency filter: 0.5 Hz; low frequency filter: 70 Hz; notch filter ON; sensitivity 100 microV/mm; 15 mm/s)*.

EEG is very useful also in AE cases associated with LGI1 and CASPR2 Abs. Despite the frequency of temporal lobe seizures in these populations, interictal epileptiform discharges (IEDs) are rare, mainly located on temporal or fronto-temporal regions with a bilateral and asynchronous distribution [60,67,68]. A striking sensitivity to hyperventilation has been observed [67,68,69,70]. Additionally, sleep can trigger IEDs onset [60]. In our experience, however, sleep is less effective in triggering epileptic abnormalities than in other forms of temporal epilepsies (unpublished observation). FBDS, a pathognomonic manifestation of anti-LGI1 encephalitis, can be EEG negative, even though some patients show an abnormal EEG pattern consisting of a large slow wave in the frontopolar, frontal or central area preceding the tonic contraction of the contralateral arm by at least 700 ms [40,71,72]. Sometimes, FBDS can appear just after an electrodecremental event [40]. Figure 2 shows some paradigmatic EEG findings in patients with AE from our experience.

## 7. Malignancy Screening

Screening for an underlying cancer should be performed in all patients with AE and needs to be guided by the associated Ab or clinical syndrome (Table 1 and Table 2) [18]. Full-body computed tomography (CT) followed, if negative, by fluorodeoxyglucose–positron emission tomography (FDG-PET) is the recommend option if the associated Ab is not yet known [18]. Specific testing can be performed in the presence of specific Abs (e.g., female patients with anti-NMDAR encephalitis should be screened for the presence of ovarian teratoma using transvaginal US and/or MRI pelvis/abdomen as first screening, while male patients with anti-Ma2 or anti-KLHL11 should be screened for the presence of testicular tumors using US and/or CT of the pelvic region; meanwhile, FDG-PET/CT needs to be considered when retroperitoneal or mediastinal germ cell tumors are suspected) [18]. When no tumor is found at first screening, it should be repeated every 4–6 months in patients with high-risk Abs (i.e., onconeural Abs) and compatible clinical phenotype. Conversely, one screening is sufficient in patients with AE harboring low-risk Abs (e.g., LGI1, CASPR2, GABAaR or GAD65). For further details, see the updated diagnostic criteria for PNS [18]. 

## 8. Challenging Clinical Scenarios: NORSE and FIRES

New-onset refractory status epilepticus (NORSE) is a rare but devastating clinical presentation characterized by the development of a refractory SE (RSE, that is a status persisting after ≥2 parenteral medications, including a benzodiazepine) without a clear acute or active structural, toxic or metabolic cause, in patients without an active epilepsy or other preexisting relevant neurological disorder [73]. It may include forms with a known etiology, typically immune-mediated or viral encephalitis. Unfortunately, most cases (up to 50%) remain cryptogenic despite an extensive diagnostic work-up [74,75]. However, clinical features do not appear to be different between cryptogenic and autoimmune cases, suggesting a similar etiology mediated by inflammatory mechanisms with elevated levels of pro-convulsant cytokines [74].

A similar condition has been also described in children, defined as febrile infection-related epilepsy syndrome (FIRES). In these cases, the presence of a febrile episode between 2 weeks and 24 h prior to the RSE onset is, by definition, required [73]. Some authors argued that NORSE and FIRES are distinct entities [76]. However, the two syndromes share many similarities, and nowadays, FIRES is considered as a subcategory of NORSE [73].

NORSE and FIRES most frequently occur in previously healthy young adults and school-aged children, although adults over 60 can also be affected. There is a female predominance in adult groups [74,77], while boys are more frequently affected in pediatric series [78]. The incidence remains unknown, but it can be estimated to be around 20% of RSE cases [79]. Clinically, a prodromal phase with fever or mild flu-like symptoms can precede seizures and SE onset by 1–14 days. Then, focal seizures with or without bilateral tonic-clonic evolution appear, rapidly evolving into SE which usually requires intensive care unit (ICU) admission [77]. 

It is difficult to make a clinical distinction between cryptogenic NORSE and AE-related NORSE. However, some other clinical features, such as cognitive impairment, behavioral changes, and sleep disturbances can point towards a specific underlying etiology [77]. For example, in a retrospective study, 11 patients with cryptogenic NORSE more frequently showed prodromal fever, symmetric brain MRI abnormalities and severe SE, and less frequent involuntary movements and psycho-behavioral symptoms than patients with NORSE associated with anti-NMDAR encephalitis [80]. The EEGs show diverse types of sporadic or periodic epileptiform discharges, which can be lateralized, bilateral independent or multifocal, often involving temporal and frontal regions. About 70% of cases show an abnormal MRI with FLAIR hypersignal involving limbic and/or neocortical areas, often bilaterally [74], with subsequent development of diffuse atrophy [81].

Most cases of FIRES and NORSE evolve to super-refractory SE (SRSE) with prolonged ICU stay and poor outcome in a substantial proportion of patients. The median duration of ICU stay in FIRES and NORSE ranges from 20 to 40 days in children [78] and 15 days in adults [74]. Mortality rate is around 12% in children [78] and increases to 16–27% in adults [74], with neurological sequelae in most survivors. 

## 9. Treatment

### Immunotherapy

When alternative etiologies are excluded and a strong suspicion of AE is present, first-line immunotherapies (including steroids, intravenous immunoglobulin (IVIG) or plasma exchange (PLEX)) should be considered early, before receiving Ab results [82]. Treatment with intravenous methylprednisolone 1 g per day for 5 consecutive days is a common approach in patients with AE, and it is the preferred strategy in scenarios known to be highly steroid-responsive, such as anti-LGI1 encephalitis [82,83], or when steroids are the recommended first-line treatment, such as in ICI-related AE [33]. Caution should be exerted when using steroid bolus in patients with uncontrolled diabetes or hypertension, or in the presence of an atypical mass lesion under consideration for brain biopsy (as in CNS lymphoma suspicion, which can sometimes mimic AE) [82]. We prefer to avoid steroids when an underlying tumor is not excluded yet, as they can hamper the detection of small tumoral lesions.

IVIG at a dose of 0.4 g/kg/day for 5 consecutive days is an alternative to corticosteroids when they are contraindicated, but should be used with caution in patients with high risk of thrombosis (e.g., elderly or oncologic patients), as they carry a thromboembolic risk [82]. Importantly, a randomized blinded trial has demonstrated the efficacy of IVIG over placebo in patients with seizures related to anti-LGI1 and anti-CASPR2 encephalitis [84]. 

**Figure 3:**
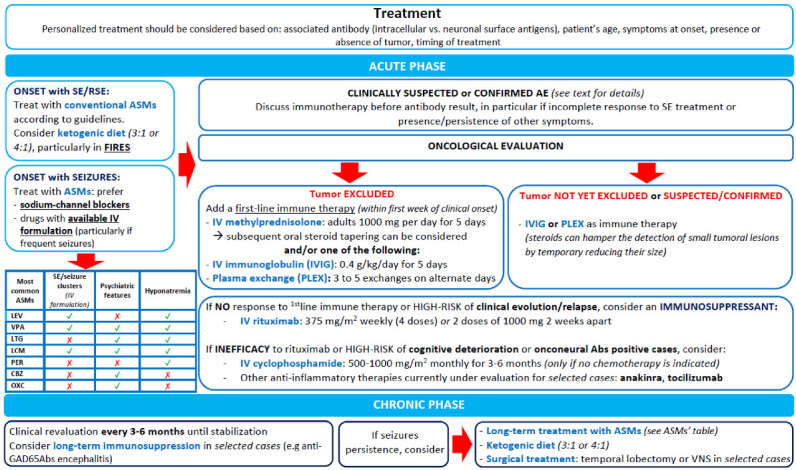
Proposed therapeutic algorithm in patients with suspected immune-mediated seizures and epilepsy. Abbreviations: AE, autoimmune encephalitis; ASMs, antiseizure medications; CBZ, carbamazepine; FIRES, febrile infection-related epilepsy syndrome; IV, intravenous; IVIG, intravenous immunoglobulin; LEV, levetiracetam; LCM, lacosamide; LTG, lamotrigine; OXC, oxcarbazepine; PLEX, plasma exchange; PER, perampanel.

When the diagnosis of AE is confirmed by a compatible clinical presentation and positive Ab testing and there is no oncological indication of chemotherapy, the use of second-line immunotherapies (i.e., rituximab or cyclophosphamide) is recommended, except for patients with mild disease course [3]. Rituximab (375 mg/m^2^ weekly over 4 weeks, or two doses of 1000 mg separated by two weeks, which is our preferred approach) is generally suggested in syndromes where the Abs were demonstrated to be pathogenic (e.g., neuronal cell surface Ab-mediated syndromes), while cyclophosphamide (600–1000 mg/m^2^) is usually proposed when a T-cell mechanism is considered to be the cause of neuronal damage (e.g., PNS due to onconeural Abs) [82], after multidisciplinary discussion with the oncologists. Other more targeted immunotherapies are promising and currently under evaluation [85]. Figure 3 summarizes the management approach we suggest for patients with AE-related seizures and epilepsy.

## 10. ASMs

ASMs are commonly prescribed in patients affected by AE, despite the characteristic drug resistance reported in this setting. The real efficacy of ASMs in AE is still unclear; particularly, it is unknown whether there is a specific type of AE that responds better to ASMs and which ASMs should be preferred [6,86]. 

Data from the literature suggest that the overall efficacy of ASMs alone in AE is low [6,86,87]. Cabezudo-Garcia and colleagues analyzed six articles, observing that 31 patients out of 139 were treated with ASMs alone from the beginning or after immunotherapy, but only 15 had some clinical benefits [86]. All the studies considered for the review had limitations, such as retrospective design and heterogeneous populations comprising both AE with definite auto-Abs and seronegative AE. However, sodium channel blockers were shown to have higher efficacy in AE, both in mono- and polytherapy [6,88,89]. Particularly, Feyissa et al. [89] and De Bruijn et al. [6] reported that a small number of patients became seizure free after treatment with sodium channel blockers, while none treated with levetiracetam had a complete seizure control. Furthermore, Feyissa et al. observed that patients harboring Abs targeting the VGKC-complex (LGI1 and CASPR2 now known as relevant antigens) were more likely to have clinical benefits from ASMs, as compared with GAD-65 positive and seronegative AE [89]. However, when using sodium channels blockers, drug-to-drug interactions must be taken into consideration, as AE patients are often treated with other agents, particularly immunosuppressant drugs, chemotherapeutic agents in paraneoplastic cases, or other ASMs in cases with drug-resistant epilepsy. Moreover, hyponatremia (e.g., due to carbamazepine or oxcarbazepine), cutaneous rash (e.g., due carbamazepine, phenytoin, or lamotrigine) and neurological side effects should be considered in the choice of ASM, especially in the case of anti-LGI1 encephalitis [3,6,87]. Some novel drugs with sodium channel blocker properties, intravenous route availability and lack of associated hyponatremia and significant drug–drug interaction (e.g., lacosamide [90]) appear to be particularly promising; however, no randomized trial or real-world study has explored their efficacy and safety in this setting yet. Psychiatric side effects should be monitored too, especially with ASMs that may affect mood and behavior, including levetiracetam, perampanel, and topiramate [3]. 

Data regarding the efficacy of ASMs on seronegative AE vary in the literature. Cabezudo-Garcia et al. reported that ASMs seem to exert a better response in case of seronegative AE [86], in contrast with Feyissa and colleagues [89]. Nevertheless, it is possible that patients diagnosed with seronegative AE might have epilepsy due to other undetermined etiologies or, alternatively, represent undiagnosed Ab-positive cases due to rarer specificities not detected by commercial kits (e.g., anti-GABAaR or anti-Glycine) [3,86]. 

Finally, long-term use of ASMs in AE is a matter of debate. According to De Bruijn et al., chronic therapy with ASMs is unnecessary in most AE patients associated with NMDAR, LGI1 and GABABR Abs [6]. Nevertheless, some patients remain at risk of relapsing seizures, especially those with EEG evidence of epileptiform activity and/or active inflammation on MRI, patients with anti-GAD65, or paraneoplastic cases associated with onconeural Abs. Therefore, continuation of ASMs may be reasonable in selected cases beyond the acute phase [3,89]. 

## 11. Treatment of Status Epilepticus

Apart from immunotherapy, the treatment of autoimmune SE does not differ from that of SE due to other etiologies. It relies on benzodiazepines as first-line treatment, intravenous ASMs as second-line and general anesthesia in case of refractory SE (RSE) [91]. There is no evidence regarding the most effective ASMs in case of autoimmune SE; nevertheless, an attempt with sodium channel blockers (e.g., lacosamide), which seem to be more effective in AE, should be considered. Perampanel, a selective antagonist of AMPA receptors, may be useful too, as AMPA receptors are over expressed during sustained epileptic firing [92]. Moreover, experimental data show that perampanel may decrease pro-inflammatory cytokines and interfere with apoptotic processes [93].

Among anesthetic drugs, ketamine has been described as effective in cases of SRSE caused by anti-NMDA encephalitis [94]. Finally, vagus nerve stimulation (VNS) has been adopted in isolated cases of SRSE caused by anti-NMDAR encephalitis [95,96]. 

## 12. Ketogenic Diets

Ketogenic diets (KD) are characterized by a reduced carbohydrate intake along with a relative increase in the proportions of fat and protein, to promote fat metabolism and to produce ketone bodies [97]. Nowadays, it is well established that KD has an anti-epileptic effect [98]. The mechanisms underlying this anti-convulsant effect are not completely understood, but it is hypothesized that ketone bodies and polyunsaturated fatty acids may act as mediators, altering neuronal excitability and conferring neuroprotection; in addition, ketone bodies counteract the assemblage of inflammasomes [98,99]. Several reports have demonstrated the efficacy of the classic KD in patients with SRSE of different etiologies [100,101]. It appears to be particularly beneficial in patients with NORSE and, specifically, FIRES [73], but this needs confirmation from controlled studies. Nabbout et al. [102] reported the data of nine patients with FIRES (with SE refractory to conventional ASMs) who received a 4:1 ratio ketogenic diet. In seven patients, KD was effective within 2 to 4 days after the onset of ketonuria, and 4 to 6 days after the onset of the diet [102]. In this setting, KD acts not only with an antiepileptic role, but also with a possible anti-inflammatory mechanism [103]. Therefore, it should be considered as a possible therapeutic option early in the treatment of FIRES.

Although several reports have studied the use of KD in the acute settings of encephalitis and SRSE, very few studies have evaluated its use in patients with epilepsy secondary to AE. Husari and colleagues described the efficacy of the modified Atkins diet (MAD), a subtype of KD, in the treatment of ten patients with post-encephalitic epilepsy [104]. After a median of 10 months of MAD, seven patients (70%) achieved ≥50% seizure reduction and in particular, three patients became seizure free [104]. Further studies are needed to explore the efficacy of KD in managing seizures in AE and its role as long-term treatment. 

## 13. Surgical Treatment

Surgery has limited indication in autoimmune epilepsies, even if patients often display MRI abnormalities such as hippocampal sclerosis (HS). In fact, only few case series assessed the role of surgery in these patients, always when ASMs and immunosuppressive treatments failed [105,106,107]. A retrospective multicenter study by Carreño et al. collected 13 patients with AE who underwent temporal lobe surgery (five even before the diagnosis of autoimmune epilepsy itself); only five of them were in Engel class I or II at the last available follow up. 

Globally, seizure outcome in AE after temporal lobe surgery is poorer than that performed in other clinical settings [108]. This result may depend on ongoing or relapsing inflammation extending beyond the resected regions, bi-temporal or insular involvement (temporal-plus epilepsy), or activation of other epileptogenic foci after surgery as a sort of network inhibition failure [108].

There are not enough reports to draw conclusions about the relationship between surgical outcome and a specific type of auto-Ab. All authors, however, agree on worse surgical outcomes in cases of patients with GAD65 Abs [106,108]. Feyissa and colleagues described four cases of anti-GAD65-associated autoimmune mesial temporal epilepsy treated with brain-responsive neurostimulation (RNS) with bilateral depth hippocampal leads [107]. Seizure outcome was good, with all patients being responders: three with 50-75% reduction of seizure frequency, and one becoming seizure-free. Furthermore, RNS electrocorticographic recordings detect electrographic seizures, and may guide immunotherapy and ASMs optimization [107]. 

In conclusion, a possible benefit of surgery should be taken into consideration in refractory epilepsy due to AE. Nevertheless, some patients may require presurgical invasive recordings to better define the epileptogenic area. Neuromodulation such as VNS and RNS may be considered in highly-select cases.

## 14. Outcome

The majority of patients with acute symptomatic seizures secondary to AE do not develop autoimmune-associated epilepsy, justifying gradual ASMs tapering after the acute phase in most seizure-free patients lacking epileptic discharges on EEG examinations [3,6]. The main problem in clinical practice is identifying the subgroup of cases in which the risk of relapse remains high. In a recent multicenter Italian study involving 263 patients with AE-related seizures, 43.7% of cases developed chronic epilepsy on long-term follow-up. The predictors of long-term epilepsy were younger age at disease onset, the detection of intracellular (onconeural or GAD65) Abs and episodes of refractory SE as well as delay in immunotherapy initiation [4]. Previous studies also found younger age at onset and immunotherapy delay as predictive factors of chronic epilepsy, while others suggested that the presence of IEDs on EEG can predict which patients will relapse [109,110]. 

## 15. Concluding Remarks and Future Perspectives

Most patients with AE develop acute symptomatic seizures, including cases of SE and RSE. A minority of them will evolve to an autoimmune-associated epilepsy, especially those with onconeural (paraneoplastic) Abs, as well as those with GAD65 Abs.

Our recommendation for minimizing seizure recurrence is to treat all AE patients with immunotherapy early over the course of the disease, and to consider ASM discontinuation in clinically stable, seizure-free cases with anti-LGI1, anti-NMDAR and anti-GABAbR lacking IEDs on EEG. In this regard, prolonged video-EEG telemetry should be preferred to avoid seizure underreporting. 

Several aspects need to be clarified in future studies addressing this relatively novel topic. First, the real impact of immune etiology in patients with epilepsy has never been investigated at the population level. Second, studies involving the identification of biomarkers (e.g., levels of cytokines, EEG patterns, etc.) to diagnose patients belonging to the most challenging scenarios (i.e., those with subtle signs of encephalitis and Ab-negative) are warranted. Third, future prospective studies should determine the ideal immunomodulatory and ASM treatment for patients, based on clinical features and Ab specificity. 

## Figures and Tables

**Table 1:** Antibodies against neuronal surface antigens frequently associated with seizures and estimated risk of epilepsy.

Autoantibody	Demographic and Oncologic Features	Clinical Specificities	Seizure Frequency	Status Epilepticus	EEG	MRI	Risk of Long-Term Epilepsy	ASMs Discontinuation Likely?
GABAbR	PNS (>50%): aged > 45 y, male predominance, smokers, associated anti-KCTD16 Abs and SCLC. Non-PNS: younger	LE	>90%	+++	>80% abnormal, mostly over the temporal lobes	Frequent MTL HS	Low	Yes
GABAaR	Non-PNS (>70%). Children can also be affected. PNS: thymoma	Encephalitis	>80%	+++	Almost invariably abnormal	Multifocal, cortical-subcortical (ADEM-like) lesions	Moderate	Unknown
NMDAR	PNS: ovarian teratomas in female aged between 12–45 y (50%). Elderly have less frequently tumors (<25%). Non-PNS: children	Encephalitis	>70%	++	90% abnormal, with 3 stages: (i) excessive beta activity 14-20 Hz; (ii) EDB; (iii) GDA	Normal or nonspecific changes. Demyelinating lesions in a subgroup	Low	Yes
LGI1	Non-PNS (>90%): elderly, male predominance. PNS: thymoma (often with concomitant anti-CASPR2 and Morvan syndrome)	LE	>75%	+	Frequent subclinical seizures, often triggered by HV, but rare IEDs. FBDS may be preceded by contralateral F slow wave	Unilateral or bilateral MTL HS (normal in 25%)	Low-Moderate	Yes
CASPR2	Non-PNS (>70%): LE PNS: Morvan syndrome and concomitant LGI1 Abs	LE, Isaac syndrome, Morvan syndrome	>60%	+	Nonspecific findings	Usually normal (bilateral MTL HS in a minority of cases)	Low	Yes

Abbreviations: Abs, antibodies; EDB, extreme delta brush; EEG, electroencephalography; F, frontal; FBDS, faciobrachial dystonic seizures; GDA, generalized delta activity; HS, hypersignal; HV, hyperventilation; IEDs, interictal epileptic discharges; KCTD16, potassium channel tetramerization domain containing 16; LE, limbic encephalitis; MRI, magnetic resonance imaging, MTL, mesial temporal lobe; PNS, paraneoplastic neurological syndromes; SCLC, small-cell lung cancer.

**Table 2:** Antibodies against intracellular antigens frequently associated with seizures and estimated risk of epilepsy.

Autoantibody	Demographic and Oncologic Features	Clinical Specificities	Seizure Frequency	Status Epilepticus	EEG	MRI	Risk of Long-Term Epilepsy	ASMs Discontinuation Likely?
Hu	PNS (85%): typically, middle-aged/elderly male smokers (tumors: mostly SCLC, less commonly NSCLC or others). ICI-triggered cases are possible. Non-PNS: younger (including children), no sex predominance	SNN, EM, and LE; some patients develop EPC	10% overall (54% in LE)	+	Nonspecific findings	Limbic involvement (10–20%)	High if the limbic system is affected	No
CV2/CRMP5	PNS (>80%): typically, middle-aged/elderly with SCLC or thymoma	EM and SNN	9–27%	−	Nonspecific findings	Limbic involvement (13–18%)	High if the limbic system is affected	No
Ma2	PNS (>75%): Young men with testicular cancer; older patients with SCLC. ICI-triggered cases are possible	LE, diencephalitis, and brainstem encephalitis	30% overall (50% in LE)	+	Unilateral or bilateral IEDs mainly involving the temporal lobes	Frequent MTL HS, hypothalamus, thalamus, brainstem HS also possible	High	No
GAD65	Non-PNS (>85%): female predominance, median age is 30 y, systemic autoimmune comorbidities. PNS: older male patients with associated Abs	LE, cerebellar ataxia, and SPS	>80%	++	Temporal lobe ictal or IEDs	MTL HS in half of the cases	High	No

Abbreviations: Abs, antibodies; EEG, electroencephalography; EM, encephalomyelitis; EPC, epilepsia partialis continua; HS, hypersignal; ICI, immune checkpoint inhibitors; IEDs, interictal epileptic discharges; LE, limbic encephalitis; MRI, magnetic resonance imaging, MTL, mesial temporal lobe; NSCLC, non-small cell lung cancer; PNS, paraneoplastic neurological syndromes; SCLC, small-cell lung cancer; SNN, sensory neuronopathy; SPS, stiff-person syndrome.
